# An Updated Review on the Use of Noninvasive Respiratory Supports in the Management of Severe Asthma Exacerbations

**DOI:** 10.3390/medicina61020328

**Published:** 2025-02-13

**Authors:** Giuseppe Cuttone, Luigi La Via, Federico Pappalardo, Massimiliano Sorbello, Daniele Salvatore Paternò, Matteo Piattoli, Cesare Gregoretti, Giovanni Misseri

**Affiliations:** 1 Department of Anaesthesia and Trauma Center, Azienda Ospedaliera “Ospedali Riuniti Villa Sofia–Cervello”, 90146 Palermo, Italy; giuseppe.cuttone@hotmail.it; 2 Department of Anaesthesia and Intensive Care 1, University Hospital Policlinico “G. Rodolico–San Marco”, 95123 Catania, Italy; luigilavia7@gmail.com; 3 Faculty of Medicine and Surgery, University of Enna “Kore”, 94100 Enna, Italy; federico.pappalardo@unikore.it (F.P.); massimiliano.sorbello@unikore.it (M.S.); 4 Policlinico “G.B. Morgagni”, 95125 Catania, Italy; 5 Department of Anaesthesia and Intensive Care, Giovanni Paolo II Hospital, 97100 Ragusa, Italy; paternomd@icloud.com; 6 Faculty of Medicine and Dentistry, Università degli Studi di Roma “La Sapienza”, 00185 Rome, Italy; m.piattoli91@gmail.com; 7 Faculty of Medicine and Surgery, Saint Camillus International University of Health and Medical Sciences “UniCamillus”, 00131 Rome, Italy; c.gregoretti@gmail.com; 8 Department of Anaesthesia and Intensive Care, Fondazione Istituto “G. Giglio” Cefalù, 90015 Palermo, Italy

**Keywords:** acute respiratory failure, ARF, asthma, CPAP, high flow nasal cannula, HFNOT, mechanical ventilation, noninvasive intermittent positive pressure ventilation, noninvasive ventilation, NIV, NPPV, status asthmaticus

## Abstract

Asthma is a reversible clinical condition characterized by airway obstruction due to bronchial smooth muscle contraction, inflammation and a hypersecretive state. Severe asthma exacerbations (SAE) may be a part of the natural history of this condition. Patients presenting with SAE are at higher risk of recurrent attacks, often nonresponsive to medical therapy and eventually requiring invasive mechanical ventilation (MV). The use of noninvasive respiratory supports (NRSs) may be beneficial in patients with SAE who are at risk of developing acute respiratory failure (ARF). However, their application is insufficiently supported by the evidence, as reports on their application in asthmatic patients are scarce and only a few retrospective studies with a limited number of participants have been published to date. This review discusses the potentialities of NRS in the treatment of SAE, with reference to the pathophysiological background and future perspectives on their use in asthma management.

## 1. Introduction

Asthma is a chronic respiratory condition characterized by airway inflammation, bronchial hyperresponsiveness and reversible airflow obstruction [1]. It affects over 300 million people worldwide and is responsible for significant morbidity, mortality and healthcare costs [2]. Severe asthma exacerbation (SAE) might be a part of the natural history of the disease; its hallmarks, including increased airway resistance, air trapping and dynamic hyperinflation, lead to increased work of breathing and potential respiratory failure [3]. SAE can cause severe acute respiratory failure (ARF), which in turn may eventually require invasive mechanical ventilation (MV). While pharmacological management, including inhaled bronchodilators and systemic corticosteroids, remain the cornerstone of asthma treatment, there has been growing interest in the use of noninvasive respiratory support (NRS) as an adjunct therapy for SAE [4,5]. The term NRS is used in reference to noninvasive intermittent positive pressure ventilation (NPPV), continuous positive airway pressure (CPAP) and high-flow nasal oxygen therapy (HFNOT) [6]. Over the past two decades, the use of NRS, especially NPPV, in acute asthma, has been the subject of numerous studies, with varying results [7]. While some trials have shown promising outcomes in terms of lung function improvement and reduced rate of hospital admissions [8,9,10], a recent Cochrane systematic review could not provide a definitive conclusion regarding the use of NRS in SAE [11]. Although a personalized approach to NRS use for SAE treatment is mandatory, it is still debated how to recognize patients who would benefit from these approaches [12]. Current guidelines [13] are unable to offer a clear recommendation on the application of NPPV in asthmatic patients, due to the uncertainty of the evidence and the paucity of research in this field. In a recent published guideline on the management of SAE, the experts were unable to recommend the use of NPPV or any other NRS strategy in SAE [14].

It has been estimated that about 10% of individuals who access emergency departments for asthma exacerbations are subsequently admitted to intensive care units (ICUs), with 2% of patients being intubated for clinical deterioration [15]. Despite ICU admission and the application of invasive MV being associated with higher mortality rates, the decision to intubate deteriorating patients experiencing SAE should not be delayed and should be based on clinical judgement. The most frequently reported complication of invasive MV in patients with SAE is hemodynamic instability manifested as hypotension, usually occurring at the initiation of ventilation and related to the decrease in systemic venous return caused by the worsening of hyperinflation. These cases may be identified early by disconnecting the patient from the ventilator; in the case of a positive hemodynamic response, invasive MV should be set with lower tidal volumes and respiratory rates, allowing adequate lung expansion and deflation. Barotrauma and tension pneumothorax are the second most frequently reported complications. Early application of NRS in SAE might avoid invasive MV and its deleterious consequences [16].

When considering the most recent estimates, NPPV use has been progressively increasing, from 3% in 1998 to 34% in 2016 [5,15], while SAE represents only 1% of the causes requiring invasive MV [17]. This update review aims to shed light on the potentialities of NRS use in the treatment of SAE, with reference to the pathophysiological background of asthma exacerbations and future perspectives on NRS application in asthma management.

## 2. Asthma Pathophysiology

Asthma is a complex, chronic respiratory disorder characterized by airway inflammation, bronchial hyperresponsiveness and reversible airflow obstruction [1].

The pathophysiology of asthma involves a complex interplay of genetic predisposition, environmental factors and immunological responses [18]. At the cellular level, various inflammatory cells, including mast cells, eosinophils, T lymphocytes (particularly Th2 cells), dendritic cells and innate lymphoid cells (ILCs), play crucial roles in orchestrating the asthmatic response [19,20]. When exposed to triggers such as allergens, viruses or irritants, these cells become activated and release a cascade of inflammatory mediators, including cytokines (e.g., IL-4, IL-5, IL-13), chemokines, leukotrienes and prostaglandins [21]. Recent research has highlighted the role of neurogenic inflammation, with sensory nerves in the airways contributing to bronchial hyperresponsiveness and inflammation through the release of neuropeptides [22]. Furthermore, the discovery of different asthma phenotypes and endotypes has revealed the complexity of this disease, with variations in underlying inflammatory patterns (e.g., Type 2 high vs. Type 2 low inflammation) and clinical presentations [23,24]. The heterogeneity in asthma pathophysiology underscores the need for personalized approaches to diagnosis and treatment [25].

The inflammatory milieu leads to structural changes in the airways, collectively known as airway remodeling, which includes epithelial damage, goblet cell hyperplasia, subepithelial fibrosis, increased smooth muscle mass and angiogenesis [26,27]. The chronic inflammation and remodeling result in bronchial hyperresponsiveness, where the airways become overly sensitive to various stimuli [28]. During SAE, this hyperresponsiveness manifests as bronchospasm, edema and mucus hypersecretion, leading to airway narrowing and increased airway resistance [3]. The narrowed airways cause air trapping, leading to severe airflow limitation, dynamic hyperinflation and intrinsic positive end-expiratory pressure (PEEP_i_). Dynamic hyperinflation may cause severe lung hyperdistention, potentially leading to barotrauma and cardiovascular collapse.

Additionally, the heterogeneous nature of airway obstruction results in ventilation-perfusion mismatch, contributing to hypoxemia [29].

The pathophysiological mechanisms described above might be contained and partially reversed through the beneficial effects of NRS use in SAE [16]: CPAP promotes a reduction of the workload of inspiratory muscle and PEEP_i_ offset, thanks to its bronchodilation effects and decrease in airway resistance, promoting clearance of bronchial secretions and reducing atelectasis formation. The use of NPPV increases spontaneous tidal volume, allowing a reduction of asthmatic patients’ inspiratory effort, a reduction in respiratory rate and an increase in expiratory time, therefore reducing the burden on the respiratory muscles [8,9,11,12,16].

## 3. Respiratory System Mechanics, Gas Exchange and Heart–Lung Interactions in Acute Asthma Exacerbations

Severe asthma exacerbations are characterized by an increase in airway resistance due to bronchoconstriction, inflammation and mucus. This results in expiratory flow limitation and dynamic hyperinflation, with the latter defined as the increase in the relaxation volume of the respiratory system at the end of a tidal expiration [30]. In normal subjects, the end-expiratory pleural pressure is negative, while the airway and alveolar pressures are zero relative to the atmosphere. When dynamic hyperinflation occurs, the alveolar pressure remains positive throughout the expiratory phase, leading to the development of intrinsic PEEP (PEEP_i_) [30]. The presence of PEEP_i_ at the initiation of the inspiratory effort and inspiratory flow requires the respiratory system to exert additional work in order to overcome PEEP_i_. In patients under NPPV, this increased inspiratory effort might lead to the “ineffective triggering” asynchrony, also known as “wasted effort” [31].

Taking in consideration the above-mentioned physiological alterations, spontaneously breathing patients with SAE experience a progressive reduction of forced expiratory volume in the first second (FEV_1_), with major lung hyperinflation, PEEP_i_ generation [32] and an unfavorable inspiratory muscle shortening, which in turn reduces the mechanical efficiency of the respiratory system, leading to dyspnea and fatigue [33]. With the worsening of airway obstruction and increased work of breathing, there is an imbalance between the production and clearance of carbon dioxide (CO_2_) and a reduced alveolar ventilation, causing the arterial partial pressure of carbon dioxide (PaCO_2_) levels to rise [33,34]. In addition to hypercapnia, the presence of airway obstruction and of a low ventilation/perfusion units hampering gas exchanges may lead to severe hypoxemia. Hypoxic vasoconstriction and changes in cardiac output may take place as a compensatory mechanism, trying to mitigate the development of hypoxia [35].

The hemodynamic alterations occur because of dynamic hyperinflation and the wide variations of intrathoracic negative pressure, which are in turn generated to overcome airway flow obstruction and PEEP_i_. The decrease in left-ventricle end-diastolic volume and in stroke volume is caused by heart–lung interactions [5,36]: while dynamic hyperinflation increases right-ventricle impedance by increasing right-ventricle afterload, the negative intrathoracic pressure increases right-ventricle preload. As a result, the septal leftward shift and flattening impairs hemodynamics. These heart–lung interactions are particularly seen in ventilated asthmatic patients, where extremely severe hyperinflation is present [37]. When applied to patients presenting SAE, NRS reduces asthmatic patients’ effort, respiratory rate and possibly lung hyperinflation, containing the large negative inspiratory swings in pleural pressure, which are the cause of compromised right and left ventricular performance [8,16,36].

## 4. Noninvasive Positive Pressure Ventilation Use in Acute Asthma Exacerbations

**Figure 1:**
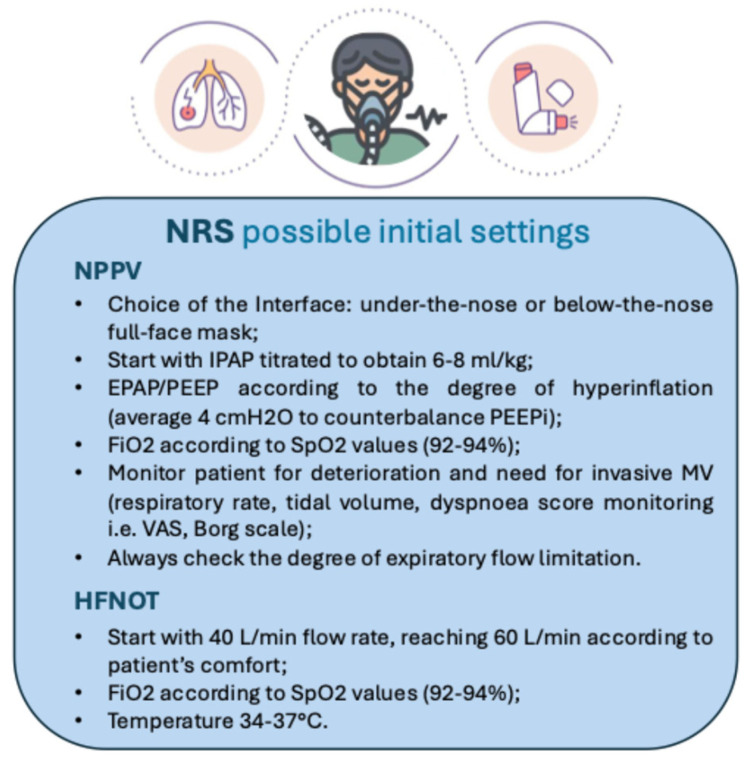
Infographic on NRS-suggested initial settings for the treatment of acute asthma exacerbations (IPAP: inspiratory positive airway pressure; EPAP: expiratory positive airway pressure, VAS: visual analogue scale) [8].

The use of NPPV in SAE remains controversial. However, its application might be considered in order to decrease the need for invasive MV and its deleterious consequences [38]. The application of NPPV with moderate levels of applied PEEP may help to counterbalance PEEP_i_, decrease the work of breathing and improve ventilation–perfusion matching [33], while recruiting collapsed alveoli [39]. Indications for NPPV generally include moderate to severe AE with persistent dyspnea, tachypnea, use of accessory muscles and signs of respiratory fatigue despite initial medical therapy. Contraindications include altered mental status, inability to protect the airway, severe hypoxemia, hemodynamic instability (secondary to severe dynamic hyperinflation, or related to other causes) and facial trauma or deformities preventing proper mask fitting [8] (Figure 1).

As bronchodilators represent a primary component for the treatment of SAE, they should not be discontinued when ARF develops and NPPV is initiated. Nebulizers and pressurized metered-dose inhalers (pMDIs) are effectively employed to deliver aerosolized medications to patients receiving NPPV [40], with no need of interface displacement and therapy interruption. Usually, there are three different configurations regarding aerosol device placement on the ventilator circuit: (1) attached to the vented mask with leak port, (2) between the leak port and ventilator on the NPPV circuit, or (3) between the leak port on the circuit and unvented mask. Evidence has shown that the optimal position of nebulizers is between the leak port and the interface: when the nebulizer is placed between the leak port and the unvented mask, the applied inspiratory pressure moves aerosol particles to the patients. While some of these escape through the leak port on the circuit during expiration, others accumulate in the tubing and are delivered in the next inspiration. In the case of vented mask use, aerosol loss occurs through the leaks, leading to a significant reduction in aerosol drug delivery during NPPV treatment [40]. Although combining aerosol therapy and NPPV did not improve aerosolized-drug pulmonary deposition, it has been demonstrated that patients receiving aerosol therapy during NPPV experienced significant improvements of pulmonary function. This might be explained by a double-positive effect: while NPPV unloads inspiratory muscles and mitigates fatigue, bronchodilators reduce resistances, leading to the potential resolution of the SAE episode.

Several studies have investigated the efficacy of NPPV in asthma exacerbations (Table 1).

In their prospective clinical study, Meduri et al. [34] investigated the use of NPPV in 17 acidotic hypercapnic patients (mean pH 7.25 ± 0.01 and mean PaCO_2_ 65 ± 2, mmHg) who failed to improve gas exchange and respiratory patterns after medical therapy. They found that NPPV was well tolerated and could be effective in correcting gas exchange abnormalities during asthma exacerbations. The mean duration of NPPV treatment was 16 ± 21 h, with a mean pressure of 18 ± 5 cm H_2_O and always less than 25 cm H_2_O. Only two patients failed NPPV and required intubation (35 min and 89 h after NPPV initiation) for PaCO_2_ worsening. Fernandez et al. [33] reported the use of NPPV in 22 subjects (67%) who failed to respond to aggressive initial management in the emergency department. When compared to patients undergoing invasive MV (11 subjects, 33%), patients treated with NPPV showed improved arterial carbon dioxide values (PaCO_2_ 89 ± 29 mmHg vs. 53 ± 13 mmHg, *p* < 0.05; pH 7.05 ± 0.21 vs. 7.28 ± 0.008, *p* < 0.05), while no differences were found in the median length of ICU stay, hospital stay and mortality. In this study, only three (14%) patients were intubated, two due to altered mental status and one due to mask intolerance.

Soroksky et al. [8] conducted a randomized controlled trial in 30 patients with SAE admitted to the emergency department, comparing bilevel positive airway pressure (BiPAP, namely inspiratory positive pressure plus PEEP) to conventional therapy. The primary end point was an increase of at least 50% in FEV_1_ as compared to baseline. Eighty percent of the patients in the BiPAP arm achieved the predetermined primary end points vs. 20% of control patients (*p* < 0.004). The authors concluded that the BiPAP group had significant improvements in FEV_1_ and peak expiratory flow (PEF) at 3 h, as well as a reduced hospitalization rate.

With the aim to clarify the effectiveness of NPPV use in SAE, Murase et al. [12] retrospectively analyzed 102 patients experiencing asthma attacks and treated in two different periods in their department (48 pre-NPPV and 54 post-NPPV introduction in clinical practice). They found that the need for intubation and invasive MV was decreased after the introduction of NPPV treatment (mean time interval between arrival and start of MV of 171.7 ± 217.9 min vs. 38.5 ± 113.8 min for NPPV, *p* < 0.05). In addition, the post-NPPV cohort presented a reduction in the duration of invasive MV or NPPV (36.9 ± 38.4 h vs. 20.3 ± 35.8 h, *p* = 0.09), and hospital stay was shortened (12.6 ± 4.2 vs. 8.4 ± 2.8 days, *p* < 0.01). The authors concluded that NPPV could be an effective treatment option for SAE, possibly decreasing the need for intubation and invasive MV in selected patients.

Similar results were found in another prospective study [41], reporting 44 patients with SAE who were randomized into a NPPV and a control group (30 and 14 patients, respectively). Patients in the NPPV group were ventilated with BiPAP (BiPAP model ST; Philips-Respironics^®^) and were further divided into two groups, high and low pressure. The authors found that the NPPV group demonstrated an improvement in FEV_1_ and that the mean percent change in FEV_1_ significantly improved after 40 min in the high-pressure group compared with that in the control group (*p* < 0.0001).

Gupta et al. [9] conducted a randomized controlled trial aiming at evaluating the efficacy of NPPV in SAE in terms of FEV_1_, intensive care unit length of stay (ICU-LOS) and hospital length of stay (hospital-LOS). The secondary end points were amelioration in arterial blood gas exchanges, pH values, respiratory rate, requirement for inhaled medications and rates of primary medical therapy failure. Patients with SAE were randomized to receive either standard therapy or NPPV, in addition to medical therapy. Fifty-three patients with SAE (42 females and 11 males, mean ± SD age of 44 ± 15 years, FEV_1_ < 30% of predicted) were randomized to NPPV (*n* = 28) or standard medical therapy (*n* = 25). In the NPPV group, the median inspiratory and expiratory airway pressures applied were 12 cm H_2_O and 5 cm H_2_O, respectively. The authors found a significant improvement in FEV_1_ and PaO_2_/F_i_O_2_ and respiratory rate in both the groups, with no significant difference between the two study arms, while pH or PaCO_2_ did not change. The number of patients who had at least 50% amelioration in FEV_1_ at 1, 2 and 4 h was not significantly greater in the NPPV arm. ICU-LOS and hospital-LOS were significantly shorter in the NPPV group. In addition, the NPPV arm required a significantly lower mean dose of inhaled bronchodilator. Four patients who did not improve with standard medical therapy improved with NPPV, and two patients in the NPPV group required intubation. The authors concluded that the addition of NPPV to standard medical therapy may speed up lung function improvement, decrease the use of inhaled bronchodilator requirements and shorten the ICU and hospital LOS.

Conversely, a Cochrane review by Lim et al. [11] found that, although NPPV may provide some benefits in terms of lung function and hospital admissions, its application in SAE is insufficiently supported by the evidence, which is of low quality and inconclusive.

In their retrospective study, Althoff et al. [42] assessed the association between NPPV and the subsequent need for invasive MV and in-hospital mortality among 53,654 patients admitted with SAE in ICUs from 682 hospitals in the United States during 2010–2017. The study found that 13,540 patients received NPPV (25.2%; 95% confidence interval [CI], 24.9–25.6%), 14,498 underwent endotracheal intubation and invasive MV (27.0%; 95% CI, 26.7–27.4%) and 1291 died (2.4%; 95% CI, 2.3–2.5%). Out of 13,540 patients receiving NPPV, 3013 patients (22.3%; 95% CI, 21.6–23.0%) required intubation, 136 of whom died (4.5%; 95% CI, 3.8–5.3%). Across all models, the use of NPPV was associated with lower odds of receiving invasive MV (adjusted generalized estimating equation odds ratio, 0.36; 95% CI, 0.32–0.40) and in-hospital mortality (odds ratio, 0.48; 95% CI 0.40–0.58). Those who received NPPV before invasive MV were more likely to have comorbid pneumonia and severe sepsis. The authors concluded that NPPV was associated with better outcomes. However, it should be used cautiously in patients with associated acute comorbidities.

In a 2-year retrospective chart-based study, Manglani et al. [38] reviewed the data of 109 patients presenting with SAE and receiving NPPV in the emergency department. The NPPV failure rate was low (9.17%), with younger patients more likely to fail NPPV, with the need of intubation and invasive MV. Interestingly, authors found that baseline asthma severity, smoking habits and body mass index (BMI) did not impact NPPV failure rate. The hospital-LOS was significantly longer in patients who failed NPPV. When considering NPPV potential side effects, no increased rate of barotrauma was found in both groups. The authors concluded that NPPV could be a safe additional tool to conventional medical therapy in the management of patients with SAE.

More recently, Briones et al. [43] investigated the use of average volume-assured pressure support (AVAPS) in ARF, including a subgroup of asthma patients. They found that AVAPS was associated with a higher success rate in hypercapnic respiratory failure compared to hypoxemic respiratory failure, suggesting that this ventilation mode may be particularly beneficial for asthma patients with hypercapnia.

Taken together, the above reported studies highlight the potential benefits of NPPV in SAE, particularly in improving lung function and reducing hospital admissions. However, they also underscore the need for further high-quality research to definitively establish the role of NPPV in asthma management and identify the patient subgroups most likely to benefit from this intervention. Undoubtedly, the key to successful NPPV application for SAE is choosing the right patient [44]. The clinical manifestations of SAE and their treatment could be described as a continuum: patients with easily controlled disease may effectively respond to medical therapies and probably do not need any NRS treatment; at the other extreme, patients with impeding ARF should be immediately intubated, and NPPV application before invasive MV could only delay adequate treatment. Between these two clinical opposite poles, patients with severe early-stage asthmatic manifestations not adequately responding to medical therapies might take advantage of a NPPV trial, possibly containing the risk for invasive MV and its potential deleterious consequences.

**Table 1:** Studies on the use of noninvasive positive pressure ventilation in acute asthma exacerbations. EPAP: expiratory positive airway pressure; ETI: endotracheal intubation; FEV1: forced expiratory volume in the first second; IPAP: inspiratory positive airway pressure; ICU: intensive care unit; MV: mechanical ventilation; NPPV: noninvasive positive pressure ventilation.

Study (First Author, Year)	Design	N. Patients Age (y) Sex	Intervention	Interfaces	Outcomes
Meduri GU et al., 1996 [34]	Prospective clinical study	17 35 ± 11 41%M/59%F	IPAP 14 ± 5 cm H_2_I EPAP 4 ± 2 cm H_2_O	NPPV face mask	Baseline at 2 h: pH 7.25 ± 0.01; PaCO_2_ 65 ± 2; PaO_2_ 315 ± 41; From 2 h to 6 h: pH 7.32 ± 0.02; PaCO_2_ 52 ± 3; PaO_2_ 403 ± 47; From 12 h to 24 h: pH 7.36 ± 0.02; PaCO_2_ 45 ± 3; PaO_2_ 367 ± 47 At 12 h: pH 7.38 ± 0.02; PaCO_2_ 45 ± 4; PaO_2_ 472 ± 67 Two patients required intubation. All patients survived. Length of hospital stay was 5 ± 4 days
Fernandez MM et al., 2001 [33]	Retrospective observational study	33 (22 NPPV vs. 11 ETI) NPPV 48 ± 21 ETI 53 ± 19 NPPV 27%M/73%F ETI 27%M/73%F	IPAP 10 cm H_2_O EPAP 5 cm H_2_O	NPPV face mask	NPPV PaCO_2_ 89 ± 29 mmHg vs. ETI PaCO_2_ 53 ± 13 mmHg; NPPV pH 7.05 ± 0.21 vs. ETI pH 7.28 ± 0.008; NPPV HCO_3_- level 22 ± 5 mmol/l vs. ETI HCO_3_-level 26 ± 6 mmol/l; No differences in the median length of ICU stay (NPPV 4.5 vs. ETI 3 days), median hospital stay (NPPV 15 vs. ETI 12 days) and mortality (NPPV 0 vs. ETI 4%)
Soroksky A et al., 2003 [8]	Prospective randomized placebo-controlled	30 (NPPV 15 vs. Stm 15) NPPV 34 ± 8 Stm 32 ± 9 NPPV 47%M/53%F Stm 53%M/47%F	IPAP 15 cm H_2_O EPAP 5 cm H_2_O	NPPV nasal mask	Primary: Increase in FEV1 ≥ 50% Secondary: Need for hospitalization Need for MV Study over 4 h
Soma T et al., 2008 [41]	Prospective randomized trial	44 (NPPV (HP) 14; NPPV (LP) 12; Stm 14) NPPV (High Pressure, HP) 37 ± 20 NPPV (Low Pressure, LP) 46 ± 14 Stm 44 ± 13 NPPV (HP) 57%M/43%F NPPV (LP) 33%M/67%F Stm 28%M/72%F	NPPV (HP) IPAP 8 cm H_2_O EPAP 6 cm H_2_O NPPV (LP) IPAP 6 cm H_2_O EPAP 4 cm H_2_O	NPPV nose or face mask	Primary: % Improvement in FEV1 Secondary: SpO_2_ Modified Borg dyspnea scale Adverse effects
Gupta D et al., 2010 [9]	Prospective randomized controlled trial	53 (NPPV 28 vs. Stm 25) 44 ± 15 21%M/79%F	IPAP min 8 cm H_2_O EPAP 4 cm H_2_O; IPAP max 20 cm H_2_O EPAP 10 cm H_2_O	NPPV oro-nasal mask	Primary: Increase in FEV1 ≥ 50% ICU and hospital stay Secondary: RR Accessory muscle use ABG values at 1, 2 and 4 h Bronchodilator usage Failure of primary therapy
Murase K et al., 2010 [12]	Retrospective cohort study	102 (pre-NPPV 48 vs. post-NPPV 54) Pre-NPPV 45 ± 20 Post-NPPV 52 ± 18 Pre-NPPV 46%M/64%F Post-NPPV 36%M/74%F	-	NPPV face mask	Pre-NPPV 9 were treated primarily by ETI; Post-NPPV 17 were treated primarily by NPPV The rate of ETI decreased in the post-NPPV period Post-NPPV: reduction in the duration of MV with ETI or NPPV (36.9 ± 38.4 h vs. 20.3 ± 35.8 h), and hospital stay was shortened (12.6 ± 4.2 vs. 8.4 ± 2.8 days)
Althoff MD et al., 2020 [42]	Retrospective cohort study	53.654 (NPPV 13.540 vs. NO-NPPV 40.114) 51 NPPV 34%M/66%F NO-NPPV 31%M/61%F	-	-	NPPV 22.3% ETI and 136 died NPPV was associated with lower odds of receiving ETI and in-hospital mortality
Briones CKH et al., 2021 [43]	Prospective clinical study	68 (2 asthma) 71 ± 19 66%M/34%F	IPAP 12 cm H_2_O EPAP 6–8 cm H_2_O	NPPV face mask	NPPV success rate was 69% and mortality rate was 20.6%

## 5. Continuous Positive Airway Pressure (CPAP) Use in Acute Asthma Exacerbations

Continuous positive airway pressure (CPAP) is another NRS modality that has been investigated for SAE treatment. The pathophysiological rational for CPAP use in SAE overlaps NPPV uses in these same conditions [45]. Positive pressure application may prevent bronchospasm induced by various stimuli [44], as reported by former studies. It has been observed that methacholine- and histamine-induced bronchospasm could be averted by application of CPAP [46]. In addition, it has been also demonstrated that externally applied PEEP may prevent exercise-induced asthma attacks [47].

The use of CPAP may be indicated in moderate to severe asthmatic patients who have persistent dyspnea and require increased work of breathing despite initial medical therapy. Contraindications are similar to NPPV, including altered mental status, inability to protect the airway, severe hypoxemia and hemodynamic instability [48].

Several studies have examined the efficacy of CPAP in SAE. Lin et al. [48] conducted a randomized controlled trial comparing CPAP to standard therapy in 40 patients with SAE. They found that the CPAP group had significant improvements in dyspnea scores, respiratory rate and peak expiratory flow rate (PEFR) at 1 h compared to the control group.

Shivaram et al. [49] investigated the effects of CPAP on 12 stable asthmatic subjects and found that it significantly reduced airway resistance and work of breathing. They concluded that CPAP could be a useful adjunct therapy in SAE.

Despite the reported results, the use of CPAP alone without pressure support in asthma has not received broad consensus; as CPAP has no pressure support, it does not possess the added benefits related to the ability of unloading respiratory muscles [44].

A systematic review by Lim et al. [11] found limited evidence to support the routine use of CPAP in acute asthma. They noted that while some studies showed improvements in lung function and physiological parameters, the overall quality of evidence was low, and more research was needed to establish the role of CPAP in asthma management.

In conclusion, more comprehensive, well-designed clinical trials are needed to explore the potential advantages of CPAP in managing SAE. Therefore, further studies are necessary to conclusively establish its effectiveness in asthma management and to identify which patient groups are most likely to benefit from this intervention. Moving forward, research efforts should concentrate on several key areas: comparing CPAP to alternative NRS strategies, determining the optimal settings and examining the long-term outcomes associated with its use in SAE.

## 6. High-Flow Oxygen Therapy (HFNOT) Use in Acute Asthma Exacerbations

High-flow nasal oxygen therapy (HFNOT) is a NRS system able to deliver up to 60 litres min^−1^ of gas at 37 °C, with an absolute humidity of 44 mg H_2_O litres^−1^ and an inspiratory oxygen fraction (F_i_O_2_) ranging from 21% to 100% [50]. The application of this device has become common in clinical practice, spanning from intensive care and emergency medicine [51,52,53] to the optimization of patients undergoing surgical procedures [6]. The set-up requires a high-pressure source of air and oxygen, an air-oxygen blender, a humidifying and heating system, a sterile water reservoir, a non-condensing circuit and an interface. There are several physiological effects supporting its use [50]: (1) improvement of muco-ciliary clearance: the air-oxygen blend is warmed and humidified, thus improving the viscosity of airway secretions and their clearance; (2) patient comfort: nasal and mucosal irritation is infrequent, due to the flow humidification and to nasal cannulas, which are well tolerated and allow minimal skin breakdown when compared to NPPV interfaces; (3) improvement of gas exchanges: the generated high flow results in an oxygen reserve that decreases CO_2_ rebreathing; the increase of one liter of high-flow rate results in a 0.7% increase [54] in end-expiratory lung volume, suggesting that this mechanism is related to the improvement of patients’ oxygenation through alveolar recruitment; (4) work of breathing reduction: with its splinting effect, HFNOT reduces resistance of the upper airways while containing the metabolic work required for inhaled gas conditioning; in addition, the respiratory rate decreases along with flow increase, without causing hypercapnia; (5) PEEP effect: HFNOT administration is associated with a range of PEEP generation, which varies according to several factors that are related to both the patient and to the device itself (type and size of nasal cannulas applied, patients’ anatomic characteristics, open or closed mouth while breathing). Most of the evidence regarding the physiological benefits of HFNOT derives from studies on the hypoxemic patients [55]. The FLORALI study [56] is a prospective randomized controlled multicenter trial including 310 patients admitted to the ICU with acute respiratory failure. The authors found that the rate of tracheal intubation (primary endpoint) was lower among patients treated with HFNOT than among those receiving conventional oxygen (COT) or NPPV (38% vs. 47% and 50%, respectively). However, these differences did not achieve statistical significance (*p* = 0.18). In a post hoc analysis including 238 severe hypoxemic patients, intubation was less likely to occur in the HFNOT group (*p* = 0.009). In addition, HFNOT use significantly improved the ventilator-free days and mortality as compared with both COT (*p* = 0.046) and NPPV (*p* = 0.006). More recently, the COVID-19 pandemic has led to several studies reporting beneficial results on the use of HFNOT in hypoxemic patients, with inconsistent results regarding its efficacy when compared to NPPV [57]. Taken together, the studies published to date indicate that HFNOT plays an important role in the treatment of de novo acute respiratory failure. However, there are still limited and contrasting data on the use of HFNOT in the treatment of SAE. In addition, most of these studies are limited to the pediatric population. In a prospective randomized controlled trial, Ballestero et al. [58] randomly assigned 62 children (age 1–14 years) to HFNOT or COT for moderate-to-severe asthma exacerbations. The authors found that 53% of the enrolled patients in the HFNOT group demonstrated a decreased pulmonary score (which is a validated measurement of asthma severity in children, assessed using a 0–3 rating scale, evaluating respiratory rate, wheezing and accessory muscles use) by at least two points when compared to the COT group. A retrospective cohort study [59] found that children treated with HFNOT had more SAEs, greater lengths of hospital stay and the need for oxygen support when compared to standard treatment. Of note, their physiological parameters improved within 3–6 h after HFNOT treatment initiation, leading to better clinical outcomes. Although tolerability and ease of use might be in favor of HFNOT application in SAE, the authors conclude that patients may experience longer ICU stay and the need to escalate to more invasive treatments. In a pilot randomized controlled trial [60] comparing the efficacy of HFNOT with COT in improving dyspnea in hypoxemic patients with SAE in the emergency department setting, the authors found improvements both in dyspnea severity and respiratory rate. At 120 min, the mean ± SD modified Borg scale (MBS) in patients receiving COT and HFNOT was 3.3 ± 2.5 and 1.4 ± 2.5, respectively (mean difference = 1.9 [95% CI = 0.2 to 3.8], *p* = 0.043). Respiratory rates were lower with HFNOT (mean difference = 4.7 [95% CI = 1.5 to 7.8], *p* = 0.001). According to Pilar et al. [61], HFNOT seems to be ineffective when compared to NPPV for SAE treatment. In this observational study, 42 children met the inclusion criteria: 20 (47.6%) received HFNOT and 22 (52.3%) received NPPV as initial respiratory support. There were no treatment failures in the NPPV group. However, eight children (40%) in the HFNOT group required escalation to NPPV. When considering LOS, there were no differences between groups. However, patients failing HFNOT presented a median length of respiratory support three-fold longer (63 h) and the hospital-LOS was also longer compared with the subjects exhibiting treatment success.

The management of SAE presentation must always be accompanied by pharmacological treatments and inhalation therapy [45,62]. Bronchodilators, anticholinergic agents and inhaled corticosteroids are the mainstay for the resolution of SAE. Inhalation drugs might be delivered during HFNOT treatment, and the optimal way to administer such therapeutic agents has yet to be identified. The location of the nebulizers and the optimal gas flow rates to be adopted have sparked several discussions. Most of the studies suggest that the upstream position of the nebulizer, before the humidifier [63], should be preferentially adopted [64]. In contrast, the placement of aerosol devices between the humidifier and the patient results in a greater aerosol deposition, which may lead to nasal cannula occlusion. As far as gas flow rate is concerned, aerosol delivery improves with lower rates, probably due to the decreased turbulence and particle impaction within the HFNOT circuit and the airways. However, the adoption of lower gas flow rates might hinder some of the physiological benefits of HFNOT. An experimental study showed that aerosol delivery was higher when the gas flow rate was set below the patient’s inspiratory flow, with a plateau effect seen at the gas flow of approximately 50% of the inspiratory flow [65]. A recent pilot observational study [66] demonstrated the feasibility and safety of the use of HFNOT and an in-line vibrating mesh nebulizer for delivering bronchodilators in patients presenting with SAE. In this study, clinical improvement of patients was demonstrated by a significant change in PEFR (147 ± 31 L/m vs. 220 ± 38 L/m; *p* < 0.001). When compared to aerosol mask nebulizers [67], HFNOT treatment did not present significant differences in terms of hospital-LOS (2.9 [IQR 2.1–3.9] vs. 3.0 [IQR 2.3–4.4] d, *p* = 0.47), pediatric ICU-LOS (1.9 [IQR 1.4–2.8] vs. 1.8 [IQR 1.5–3.0] d, *p* = 0.92) or time to MPIS (modified pulmonary index score) < 6 (1.0 [IQR 0.6–1.6] vs. 1.3 [IQR 0.8–1.9) d, *p* = 0.09). Median time on continuous albuterol was shorter in the HFNOT group compared to the aerosol mask group (1.0 [IQR 0.7–1.8] vs. 1.5 [IQR 0.9–2.3] d, *p* = 0.048).

The inhalation of heated and humidified gases has been proven to be of particular importance in patients suffering from asthma; the inhalation of dry and cold gases is an irritative factor, which causes airway inflammation, damages the bronchial epithelium and hinders mucus clearance, while worsening bronchial hyperresponsiveness [68]. In addition, the high flow rates reached by HFNOT meet patients’ respiratory requirements, with a positive effect on respiratory gas exchanges, work of breathing and respiratory rate [69].

In conclusion, HFNOT should be considered a feasible and safe alternative among NRSs in the treatment of SAE. However, patients should always be strictly followed in order to avoid deterioration of their respiratory conditions and delayed escalation to NPPV or invasive MV.

## 7. Future Directions

Most of the studies published to date are retrospective and often of limited quality. Since retrospective studies depend on the review of charts that were not originally designed to collect data for research, some information is bound to be missing. Selection and recall biases also affect the results and reasons for differences in treatment between patients and lost follow-ups often cannot be ascertained and may lead to bias. Therefore, large-scale, multicenter randomized controlled trials are needed to definitively establish the efficacy of NRS in different asthma phenotypes and severity levels. Future research should focus on identifying the specific patient subgroups that are most likely to benefit from NRS [6], potentially through the development of predictive models or biomarkers. The optimization of NPPV protocols, including the ideal timing of initiation, the duration of use and optimal interfaces and weaning strategies, is crucial for maximizing benefits while minimizing potential complications [70,71].

Future studies should also explore the application of novel NPPV modes, such as adaptive servo-ventilation or volume-assured pressure support, which may yield outcome improvement in asthma patients. The integration of NPPV with emerging technologies, like artificial intelligence for automated adjustments or remote monitoring capabilities, could enhance its effectiveness and accessibility [72,73]. Pediatric-specific studies are needed to establish clear guidelines for ventilation strategies in children with SAE. With regards to HFNOT and CPAP, further studies should be carried in order to confirm their effectiveness in attenuating bronchial hyperresponsiveness and improving bronchodilation and inhaled bronchodilators administration.

Long-term follow-up studies should assess the impact of NRS on asthma control, quality of life and healthcare utilization. Finally, cost-effectiveness analyses across different healthcare settings will be vital in informing policy decisions regarding the widespread implementation of NRS in asthma management. 

## 8. Conclusions

Over the last 10 years, the use of several NRSs has gained wide acceptance for various indications. The use of NRSs in SAE has emerged as a promising adjunct therapy with potential benefits, including reduced work of breathing, improved gas exchange and avoidance of intubation. Current evidence suggests that NRS can improve lung function and reduce hospital admissions in selected patients with SAE, particularly when used in early stages and in conjunction with standard medical therapy. Therefore, it is possible to conclude that under appropriate circumstances and experienced professional supervision, NRS use can be extended to diseases such as asthma, which was previously considered as a contraindication. However, the overall quality of evidence remains limited, and careful patient selection is crucial. Larger, well-designed studies are needed to definitively establish the role of NRS in asthma management, optimize treatment protocols and identify the patients most likely to benefit from this intervention.

## Data Availability

Data supporting the conclusions of this article will be made available by the authors on request.

## References

[1] Global Initiative for Asthma—GINA 2024 GINA Main Report. https://ginasthma.org/2024-report/.

[2] Vos T., Lim S.S., Abbafati C., Abbas K.M., Abbasi M., Abbasifard M., Abbasi-kangevari M., Abbastabar H., Abd-Allah F., Abdelalim A. (2020). Global burden of 369 diseases and injuries in 204 countries and territories, 1990–2019: A systematic analysis for the Global Burden of Disease Study 2019. Lancet.

[3] Papiris S., Kotanidou A., Malagari K., Roussos C. (2001). Clinical review: Severe asthma. Crit. Care Lond. Engl..

[4] Brenner B., Corbridge T., Kazzi A. (2009). Intubation and mechanical ventilation of the asthmatic patient in respiratory failure. J. Allergy Clin. Immunol..

[5] Demoule A., Brochard L., Dres M., Heunks L., Jubran A., Laghi F., Mekontso-Dessap A., Nava S., Ouanes-Besbes L., Peñuelas O. (2020). How to ventilate obstructive and asthmatic patients. Intensive Care Med..

[6] Misseri G., Frassanito L., Simonte R., Rosà T., Grieco D.L., Piersanti A., De Robertis E., Gregoretti C. (2023). Personalized Noninvasive Respiratory Support in the Perioperative Setting: State of the Art and Future Perspectives. J. Pers. Med..

[7] Leatherman J. (2015). Mechanical Ventilation for Severe Asthma. Chest.

[8] Soroksky A., Stav D., Shpirer I. (2003). A pilot prospective, randomized, placebo-controlled trial of bilevel positive airway pressure in acute asthmatic attack. Chest.

[9] Gupta D., Nath A., Agarwal R., Behera D. (2010). A prospective randomized controlled trial on the efficacy of noninvasive ventilation in severe acute asthma. Respir. Care.

[10] Gregoretti C., Pisani L., Cortegiani A., Ranieri V.M. (2015). Noninvasive ventilation in critically ill patients. Crit. Care Clin..

[11] Lim W.J., Mohammed Akram R., Carson K.V., Mysore S., Labiszewski N.A., Wedzicha J.A., Rowe B.H., Smith B.J. (2012). Non-invasive positive pressure ventilation for treatment of respiratory failure due to severe acute exacerbations of asthma. Cochrane Database Syst. Rev..

[12] Murase K., Tomii K., Chin K., Tsuboi T., Sakurai A., Tachikawa R., Harada Y., Takeshima Y., Hayashi M., Ishihara K. (2010). The use of non-invasive ventilation for life-threatening asthma attacks: Changes in the need for intubation. Respirol. Carlton Vic..

[13] Rochwerg B., Brochard L., Elliott M.W., Hess D., Hill N.S., Nava S., Navalesi P., Antonelli M., Brozek J., Conti G. (2017). Official ERS/ATS clinical practice guidelines: Noninvasive ventilation for acute respiratory failure. Eur. Respir. J..

[14] Le Conte P., Terzi N., Mortamet G., Abroug F., Carteaux G., Charasse C., Chauvin A., Combes X., Dauger S., Demoule A. (2019). Management of severe asthma exacerbation: Guidelines from the Société Française de Médecine d’Urgence, the Société de Réanimation de Langue Française and the French Group for Pediatric Intensive Care and Emergencies. Ann. Intensive Care.

[15] Pendergraft T.B., Stanford R.H., Beasley R., Stempel D.A., Roberts C., McLaughlin T. (2004). Rates and characteristics of intensive care unit admissions and intubations among asthma-related hospitalizations. Ann. Allergy Asthma Immunol. Off Publ. Am. Coll. Allergy Asthma Immunol..

[16] La Via L., Cuttone G., Misseri G., Sorbello M., Pappalardo F., Maniaci A., Duarte-Medrano G., Nuño-Lámbarri N., Zanza C., Gregoretti C. (2025). The use of noninvasive positive pressure ventilation for severe asthma: A systematic review and meta-analysis of randomized controlled trials with trial sequential analysis. Expert Rev. Respir. Med..

[17] Peñuelas O., Muriel A., Abraira V., Frutos-Vivar F., Mancebo J., Raymondos K., Du B., Thille A.W., Ríos F., González M. (2020). Inter-country variability over time in the mortality of mechanically ventilated patients. Intensive Care Med..

[18] Moffatt M.F., Gut I.G., Demenais F., Strachan D.P., Bouzigon E., Heath S., von Mutius E., Farrall M., Lathrop M., William O.C.M. (2010). A large-scale, consortium-based genomewide association study of asthma. N. Engl. J. Med..

[19] Lambrecht B.N., Hammad H. (2015). The immunology of asthma. Nat. Immunol..

[20] Holgate S.T. (2012). Innate and adaptive immune responses in asthma. Nat. Med..

[21] Barnes P.J. (2008). The cytokine network in asthma and chronic obstructive pulmonary disease. J. Clin. Investig..

[22] Mazzone S.B., Undem B.J. (2016). Vagal Afferent Innervation of the Airways in Health and Disease. Physiol. Rev..

[23] Wenzel S.E. (2012). Asthma phenotypes: The evolution from clinical to molecular approaches. Nat. Med..

[24] Kuruvilla M.E., Lee F.E.H., Lee G.B. (2019). Understanding Asthma Phenotypes, Endotypes, and Mechanisms of Disease. Clin. Rev. Allergy Immunol..

[25] Agache I., Akdis C.A. (2019). Precision medicine and phenotypes, endotypes, genotypes, regiotypes, and theratypes of allergic diseases. J. Clin. Investig..

[26] Bergeron C., Tulic M.K., Hamid Q. (2010). Airway remodelling in asthma: From benchside to clinical practice. Can. Respir. J..

[27] Fehrenbach H., Wagner C., Wegmann M. (2017). Airway remodeling in asthma: What really matters. Cell Tissue Res..

[28] Cockcroft D.W., Davis B.E. (2006). Mechanisms of airway hyperresponsiveness. J. Allergy Clin. Immunol..

[29] Wagner P.D. (1992). Ventilation-perfusion matching during exercise. Chest.

[30] Vassilakopoulos T., Toumpanakis D., Mancebo J. (2020). What’s new about pulmonary hyperinflation in mechanically ventilated critical patients. Intensive Care Med..

[31] Smith T.C., Marini J.J. (1988). Impact of PEEP on lung mechanics and work of breathing in severe airflow obstruction. J. Appl. Physiol..

[32] Tobin M.J. (2004). Asthma, airway biology, and nasal disorders in AJRCCM 2003. Am. J. Respir. Crit. Care Med..

[33] Fernández M.M., Villagrá A., Blanch L., Fernández R. (2001). Non-invasive mechanical ventilation in status asthmaticus. Intensive Care Med..

[34] Meduri G.U., Cook T.R., Turner R.E., Cohen M., Leeper K.V. (1996). Noninvasive positive pressure ventilation in status asthmaticus. Chest.

[35] Young I.H., Bye P.T.P. (2011). Gas exchange in disease: Asthma, chronic obstructive pulmonary disease, cystic fibrosis, and interstitial lung disease. Compr. Physiol..

[36] Cheyne W.S., Gelinas J.C., Eves N.D. (2018). Hemodynamic effects of incremental lung hyperinflation. Am. J. Physiol. Heart Circ. Physiol..

[37] Corbridge T.C., Hall J.B. (1995). The assessment and management of adults with status asthmaticus. Am. J. Respir. Crit. Care Med..

[38] Manglani R., Landaeta M., Maldonado M., Hoge G., Basir R., Menon V. (2021). The use of non-invasive ventilation in asthma exacerbation—A two year retrospective analysis of outcomes. J. Community Hosp. Intern. Med. Perspect..

[39] Nava S., Hill N. (2009). Non-invasive ventilation in acute respiratory failure. Lancet Lond. Engl..

[40] Ari A. (2019). How to optimize aerosol drug delivery during noninvasive ventilation: What to use, how to use it, and why?. Eurasian J. Pulmonol..

[41] Soma T., Hino M., Kida K., Kudoh S. (2008). A prospective and randomized study for improvement of acute asthma by non-invasive positive pressure ventilation (NPPV). Intern. Med..

[42] Althoff M.D., Holguin F., Yang F., Grunwald G.K., Moss M., Vandivier R.W., Ho P.M., Kiser T.H., Burnham E.L. (2020). Noninvasive Ventilation Use in Critically Ill Patients with Acute Asthma Exacerbations. Am. J. Respir. Crit. Care Med..

[43] Briones Claudett K.H., Rodriguez A.E., Briones Claudett M.H., Tejada M.P., del Pilar Cabrera Baños M., Jorge D.N., Bermeo B., Grunauer M. (2021). Non-invasive mechanical ventilation with average volume-assured pressure support. Results according to the aetiology of acute respiratory failure. Anaesthesiol. Intensive Ther..

[44] Soroksky A., Klinowski E., Ilgyev E., Mizrachi A., Miller A., Yehuda T.M.B., Shpirer I., Leonov Y. (2010). Noninvasive positive pressure ventilation in acute asthmatic attack. Eur. Respir. Rev..

[45] British Thoracic Society (2014). Scottish Intercollegiate Guidelines Network. British guideline on the management of asthma. Thorax.

[46] Martin J.G., Shore S., Engel L.A. (1982). Effect of continuous positive airway pressure on respiratory mechanics and pattern of breathing in induced asthma. Am. Rev. Respir. Dis..

[47] Wilson B.A., Jackson P.J., Evans J. (1981). Effects of positive end-expiratory pressure breathing on exercise-induced asthma. Int. J. Sports Med..

[48] Lin H.C., Wang C.H., Yang C.T., Huang T.J., Yu C.T., Shieh W.B., Kuo H.P. (1995). Effect of nasal continuous positive airway pressure on methacholine-induced bronchoconstriction. Respir. Med..

[49] Shivaram U., Miro A.M., Cash M.E., Finch P.J., Heurich A.E., Kamholz S.L. (1993). Cardiopulmonary responses to continuous positive airway pressure in acute asthma. J. Crit. Care.

[50] Renda T., Corrado A., Iskandar G., Pelaia G., Abdalla K., Navalesi P. (2018). High-flow nasal oxygen therapy in intensive care and anaesthesia. Br. J. Anaesth..

[51] Roca O., Riera J., Torres F., Masclans J.R. (2010). High-flow oxygen therapy in acute respiratory failure. Respir. Care.

[52] Grieco D.L., Maggiore S.M., Roca O., Spinelli E., Patel B.K., Thille A.W., Barbas C.S.V., de Acilu M.G., Cutuli S.L., Bongiovanni P. (2021). Non-invasive ventilatory support and high-flow nasal oxygen as first-line treatment of acute hypoxemic respiratory failure and ARDS. Intensive Care Med..

[53] Maggiore S.M., Idone F.A., Vaschetto R., Festa R., Cataldo A., Antonicelli F., Montini L., De Gaetano A., Navalesi P., Antonelli M. (2014). Nasal high-flow versus Venturi mask oxygen therapy after extubation. Effects on oxygenation, comfort, and clinical outcome. Am. J. Respir. Crit. Care Med..

[54] Corley A., Caruana L.R., Barnett A.G., Tronstad O., Fraser J.F. (2011). Oxygen delivery through high-flow nasal cannulae increase end-expiratory lung volume and reduce respiratory rate in post-cardiac surgical patients. Br. J. Anaesth..

[55] Ricard J.D., Roca O., Lemiale V., Corley A., Braunlich J., Jones P., Kang B.J., Lellouche F., Nava S., Rittayamai N. (2020). Use of nasal high flow oxygen during acute respiratory failure. Intensive Care Med..

[56] Frat J.P., Thille A.W., Mercat A., Girault C., Ragot S., Perbet S., Prat G., Boulain T., Morawiec E., Cottereau A. (2015). High-Flow Oxygen through Nasal Cannula in Acute Hypoxemic Respiratory Failure. N. Engl. J. Med..

[57] Genecand L., Agoritsas T., Ehrensperger C., Kharat A., Marti C. (2022). High-flow nasal oxygen in acute hypoxemic respiratory failure: A narrative review of the evidence before and after the COVID-19 pandemic. Front. Med..

[58] Ballestero Y., De Pedro J., Portillo N., Martinez-Mugica O., Arana-Arri E., Benito J. (2018). Pilot Clinical Trial of High-Flow Oxygen Therapy in Children with Asthma in the Emergency Service. J. Pediatr..

[59] Martínez F.G., Sánchez M.I.G., Del Castillo B.T., Moreno J.P., Muñoz M.M., Jiménez C.R., Fernández R.R. (2019). Treatment with high-flow oxygen therapy in asthma exacerbations in a paediatric hospital ward: Experience from 2012 to 2016. An. Pediatr..

[60] Ruangsomboon O., Limsuwat C., Praphruetkit N., Monsomboon A., Chakorn T. (2021). Nasal High-flow Oxygen Versus Conventional Oxygen Therapy for Acute Severe Asthma Patients: A Pilot Randomized Controlled Trial. Acad. Emerg. Med..

[61] Pilar J., Modesto I Alapont V., Lopez-Fernandez Y.M., Lopez-Macias O., Garcia-Urabayen D., Amores-Hernandez I. (2017). High-flow nasal cannula therapy versus non-invasive ventilation in children with severe acute asthma exacerbation: An observational cohort study. Med. Intensiva..

[62] Chao K.Y., Chien Y.H., Mu S.C. (2021). High-flow nasal cannula in children with asthma exacerbation: A review of current evidence. Paediatr. Respir. Rev..

[63] Ari A., Atalay O., Harwood R., Sheard M., Aljamhan E., Fink J. (2010). Influence of nebulizer type, position, and bias flow on aerosol drug delivery in simulated pediatric and adult lung models during mechanical ventilation. Respir. Care.

[64] Li J., Tu M., Yang L., Jing G., Fink J., Burtin C., de Andrade A.D., Gong L., Xie L., Ehrmann S. (2021). Worldwide clinical practice of high-flow nasal cannula and concomitant aerosol therapy in the adult ICU setting. Respir. Care.

[65] Li J., Gong L., Fink J. (2019). The ratio of nasal cannula gas flow to patient inspiratory flow on trans-nasal pulmonary aerosol delivery for adults: An in vitro study. Pharmaceutics.

[66] Colaianni-Alfonso N., Toledo A., Montiel G., Castro-Sayat M., Crimi C., Vetrugno L. (2024). High-Flow Nasal Cannula and in-line aerosolised bronchodilator delivery during severe exacerbation of asthma in adults: A feasibility Observational Study. Anaesth. Crit. Care Pain Med..

[67] Gates R.M., Haynes K.E., Rehder K.J., Zimmerman K.O., Rotta A.T., Miller A.G. (2021). High-Flow Nasal Cannula in Pediatric Critical Asthma. Respir. Care.

[68] Gross J.L., Park G.R. (2012). Humidification of inspired gases during mechanical ventilation. Minerva. Anestesiol..

[69] Geng W., Batu W., You S., Tong Z., He H. (2020). High-Flow Nasal Cannula: A Promising Oxygen Therapy for Patients with Severe Bronchial Asthma Complicated with Respiratory Failure. Can. Respir. J..

[70] Vaschetto R., Gregoretti C., Scotti L., De Vita N., Carlucci A., Cortegiani A., Crimi C., Mattei A., Scala R., Rocca E. (2023). A pragmatic, open-label, multi-center, randomized controlled clinical trial on the rotational use of interfaces vs standard of care in patients treated with noninvasive positive pressure ventilation for acute hypercapnic respiratory failure: The ROTAtional-USE of interface STUDY (ROTA-USE STUDY). Trials.

[71] Pierucci P., Portacci A., Carpagnano G.E., Banfi P., Crimi C., Misseri G., Gregoretti C. (2022). The right interface for the right patient in noninvasive ventilation: A systematic review. Expert Rev. Respir. Med..

[72] Vitale F., Misseri G., Ingoglia G., Bonanno G., Gregoretti C., Giarratano A., Cortegiani A. (2020). Fake news and patient-family-physician interaction in critical care: Concepts, beliefs and potential countermeasures. Anaesthesiol. Intensive Ther..

[73] Misseri G., Piattoli M., Cuttone G., Gregoretti C., Bignami E.G. (2024). Artificial Intelligence for Mechanical Ventilation: A Transformative Shift in Critical Care. Ther. Adv. Pulm. Crit. Care Med..

